# Immobilization of Jagged1 Enhances Vascular Smooth Muscle Cells Maturation by Activating the Notch Pathway

**DOI:** 10.3390/cells10082089

**Published:** 2021-08-14

**Authors:** Kathleen Zohorsky, Shigang Lin, Kibret Mequanint

**Affiliations:** 1 School of Biomedical Engineering, University of Western Ontario, 1151 Richmond Street, London, ON N6A 5B9, Canada; kzohorsk@uwo.ca; 2 Department of Chemical and Biochemical Engineering, University of Western Ontario, 1151 Richmond Street, London, ON N6A 5B9, Canada; slin45@uwo.ca

**Keywords:** Notch signaling, Jagged1, smooth muscle cells, differentiation, progenitor cells

## Abstract

In Notch signaling, the Jagged1-Notch3 ligand-receptor pairing is implicated for regulating the phenotype maturity of vascular smooth muscle cells. However, less is known about the role of Jagged1 presentation strategy in this regulation. In this study, we used bead-immobilized Jagged1 to direct phenotype control of primary human coronary artery smooth muscle cells (HCASMC), and to differentiate embryonic multipotent mesenchymal progenitor (10T1/2) cell towards a vascular lineage. This Jagged1 presentation strategy was sufficient to activate the Notch transcription factor HES1 and induce early-stage contractile markers, including smooth muscle α-actin and calponin in HCASMCs. Bead-bound Jagged1 was unable to regulate the late-stage markers myosin heavy chain and smoothelin; however, serum starvation and TGFβ1 were used to achieve a fully contractile smooth muscle cell. When progenitor 10T1/2 cells were used for Notch3 signaling, pre-differentiation with TGFβ1 was required for a robust Jagged1 specific response, suggesting a SMC lineage commitment was necessary to direct SMC differentiation and maturity. The presence of a magnetic tension force to the ligand-receptor complex was evaluated for signaling efficacy. Magnetic pulling forces downregulated HES1 and smooth muscle α-actin in both HCASMCs and progenitor 10T1/2 cells. Taken together, this study demonstrated that (i) bead-bound Jagged1 was sufficient to activate Notch3 and promote SMC differentiation/maturation and (ii) magnetic pulling forces did not activate Notch3, suggesting the bead alone was able to provide necessary clustering or traction forces for Notch activation. Notch is highly context-dependent; therefore, these findings provide insights to improve biomaterial-driven Jagged1 control of SMC behavior.

## 1. Introduction

Notch signaling is critical in vascular development including angiogenesis, vasculogenesis, tip-stalk cell patterning and arterial specification [1,2,3]. Furthermore, irregular Notch ligand-receptor patterning in vascular tissues has been prevalent after injury [4]. In the vasculature, Notch has also been associated with disease states and disease progression. Atherosclerosis has been directly linked to Notch dysregulation, associated with phenotype switching and abnormal distribution profiles in plaque [5,6,7]. Thus, Notch signaling has been proposed as a novel therapeutic target for the vasculature and smooth muscle cell (SMC) control. Notch is an evolutionarily conserved pathway that seems simple at first glance; however, it is extremely diverse in its effect. Notch is regulated by cell-to-cell interactions with transmembrane proteins of the Jagged (Jagged1, Jagged2) or Delta-like (Dll, Dll3, Dll4) family of ligands that interact with transmembrane Notch receptors (Notch 1–4). Unlike other enzymatically amplified signaling pathways, Notch signaling relies on ligand-receptor stoichiometric interactions, and imbalances may inhibit or dysregulate the pathway [8]. Different Notch ligand-receptor binding combinations result in distinct roles in tissue development and repair, indicating the context dependent behavior in various cell and tissue types. The Jagged1-Notch3 pairing is especially important in regulating vascular tone, differentiation, and phenotype control [9,10,11,12].

Modulation of Notch signaling can be challenging as there are many complex integrating factors. Notch can be controlled by delivery of Notch components, spatial cues, temporal cues, the surrounding microenvironment (e.g., flow systems, strain application) [13] and signaling cross-talks (e.g., TGFβ, BMP) [14,15]. Additionally, the Notch “pulling model” suggests that a force is needed for Notch activation and that the Notch regulatory region (NRR) acts as a force sensor that can be unfolded by a threshold force generated across the ligand/receptor bridge. This is hypothesized to be provided by endocytosis in the signal sending cell [8,16], but is controversial, and other factors including clustering may be responsible for Notch activation [17,18,19]. Force measurements using Dll1 immobilized tension gauge tethers (TGT) have been used to quantify this force [20,21]. Additionally, force application via magnetic tweezers has provided a pulling force on Dll4-tethered beads bound to Notch receptors, proven necessary for signal activation [22]. The investigation into force has not been extended beyond Dll1 and Dll4 ligands. Consequently, the effects of molecular force application are unclear for Jagged-type ligands.

The role of endothelial-driven Jagged1 signaling has been extensively studied [23,24,25], but in damaged or diseased vessels, this signal is often lost or missing. Given the heterotypic and contact-dependent role of Notch, loss of Jagged1 can cause dysregulation. Therefore, there is an increasing need in regenerative medicine and tissue engineering approaches to create functional cell-directing biomaterials to recover lost function within dysfunctional tissues. Integration of these complex cellular processes discussed above into biomaterial-based systems is necessary, and the delivery strategy for signal control of Jagged1 is critical.

Previous studies have demonstrated a requirement for Jagged1 immobilization to a material to activate Notch signaling [26,27,28,29,30]. For example, Jagged1 immobilized to an osteoconductive polymer was needed to control osteoblast differentiation [31] and Jagged1 immobilized hydrogels created a perivascular switch in mesenchymal stem cell fate [32]. In terms of the presentation strategy, indirect binding of a recombinant Jagged1/Fc to a substrate was more effective than direct binding [33]. In view of these considerations, understanding Notch signaling and the factors that influence Notch modulation are important to improve vascular therapies or create vascular substitutes that more accurately mimic native tissues. Thus, we aim to develop a strategy to deliver Jagged1 to control vascular tone and vascular differentiation in the absence of an endothelial cell (EC) signal.

## 2. Materials and Methods

### 2.1. Smooth Muscle Cell and 10T1/2 Cell Culture

Human coronary artery smooth muscle cells (HCASMCs) (passages 4–11) were maintained in Smooth Muscle Cell Growth Medium 2 BulletKit (SmGM-2, Lonza, Walkersville, MD, USA). Non-differentiated embryonic multipotent mesenchymal progenitor cell line (10T1/2 cells) (ATCC, Gaithersberg, MD, USA) were maintained in Dulbecco’s modified Eagle’s medium (DMEM, Thermo Fisher, Waltham, MA, USA) supplemented with 5% fetal bovine serum (FBS) (Thermo Fisher, Waltham, MA, USA) and 1% penicillin/streptomycin by volume. Cells were pre-differentiated towards a vascular lineage with transforming growth factor beta-1 (TGFβ1, Abcam, Cambridge, MA, USA) for a period of 3 days. Both undifferentiated and pre-differentiated 10T1/2 cells were used. All cells were maintained in a humidified incubator at 5% CO_2_ and 37 °C and passaged when they reached 80% confluence. Media changes were regularly performed according to the cell-specific supplier’s recommendations.

### 2.2. HCASMC Serum Starvation

Cover slides were coated with 0.1% gelatin for 1 h at 37 °C. HCASMCs were seeded at 2500 cells/cm^2^, then treated when cells reached 50% confluency. Cells were cultured in SmGM-2 as a control, in serum-free Dulbecco’s modified Eagles Medium (DMEM, Thermo Fisher, Waltham, MA, USA), or serum-free DMEM with soluble TGFβ1 (2 ng/mL). After 72 h, cells were fixed, stained, and imaged with a confocal microscope.

### 2.3. Jagged1/Fc Protein Bead Immobilization and Notch-Directed SMC Response

**Figure 1:**
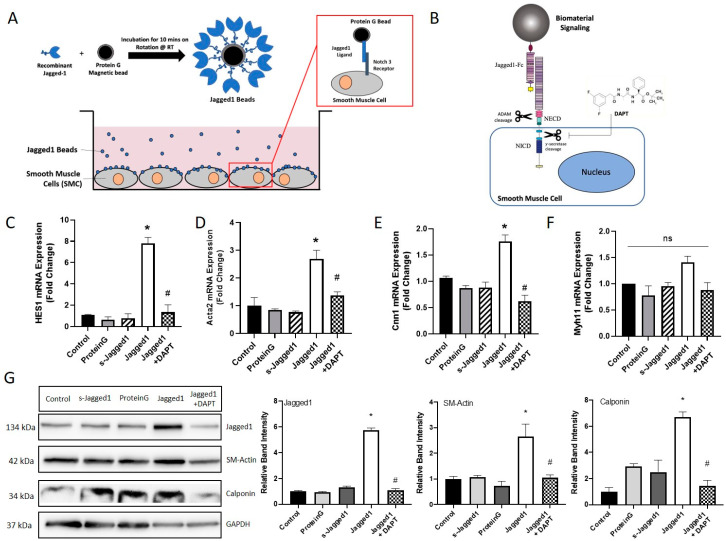
Bead-bound Jagged1 directs HCASMC contractile gene and protein expression. (**A**) Schematic drawing of Jagged1 affinity immobilization and bead-bound Jagged1 co-culture with HCASMCs. (**B**) Schematic drawing of DAPT inhibition of the γ-secretase intracellular cleavage, stopping the NICD from translocating to the nucleus. The effect of soluble and bead-bound Jagged1 was investigated, and a Notch-specific response was determined using 10 μM DAPT as Notch inhibitor. The control cells are untreated HCASMCs cultured in SmGM-2. (**C**–**F**) Gene expression of *HES1*, *Acta2*, *Cnn1,* and *Myh11*. (**G**) Representative Western blots of Jagged1, SM-Actin, and Calponin and the corresponding quantified band intensities. All data are normalized to the control and presented as the mean ± SD, *n* = 3. * indicates significance from the control, and # indicates significant downregulation from the Jagged1 treatment group at *p* < 0.05, ns indicates no statistical significance.

Protein G Dynabeads^TM^ (Thermo Fisher, Ottawa, ON, Canada) were washed with phosphate-buffered saline (PBS; pH 7.4, 0.02% Tween). 2.5 μg of human Jagged1/Fc chimera protein (R&D Systems, Minneapolis, MN, USA) (reconstituted in PBS at 200 μg/mL) was added to the bead suspension per mL of cell culture media, and incubated for 10 min under rotation at room temperature (Scheme shown in Figure 1A). Jagged1 beads were washed with PBS to remove loosely bound and soluble Jagged1. Jagged1 beads were resuspended in PBS and added to cell cultures at a concentration of 200 beads/cell. HCASMCs, undifferentiated 10T1/2 and pre-differentiated 10T1/2 cells were seeded on 24-well plates or on fibronectin (FN) coated cover slides and incubated for 48 h to allow cell attachment. Cells were treated with Protein G beads (200 beads/cell), soluble Jagged1 (2.5 μg/mL), or Jagged1 immobilized beads (2.5 μg/mL, 200 beads/cell), to determine the effects of Jagged1 delivery on SMC response. Pre-treatment of cells overnight with 10 μM DAPT (Sigma-Aldrich, Milwaukee, WI, USA) was also added to cultures to inhibit Notch signaling. After overnight incubation, the media was exchanged, then cells were treated with Jagged1 beads (2.5 μg/mL, 200 beads/cell) in combination with 10 μM DAPT. Cells cultured alone were used as controls for these experiments. Cells were analyzed by RT-qPCR, western blot, and immunofluorescence microscopy.

### 2.4. HCASMC-Jagged1-TGFβ1 Cross-Talk

Cells seeded on 24-well plates at a density of 50,000 cells/well were cultured for 48 h to attach and spread. Cells were treated with bead-immobilized Jagged1 (2.5 μg/mL, 200 beads/cell), and TGFβ1 (2 ng/mL) and Notch signaling was inhibited with DAPT (10 μM). Combination treatments were used to explore cross-talk and synergistic actions. Gene and protein expression levels were measured using RT-qPCR and immunofluorescence microscopy.

### 2.5. Jagged1-Notch3 Magnetic Tweezer Assay

HCASMCs or pre-differentiated 10T1/2 cells were seeded on flat-bottom 96-well plates at a density of 10,000 cells/well for 48 h. Bead-immobilized Jagged1 (2.5 μg/mL, 200 beads/cell) were added to the cultures and left overnight to allow for receptor-ligand binding. A 96-cylindrical magnet plate (Alpaqua Engineering, Beverly, MA, USA) (shown in Figure 5A) was positioned over the 96-well plate to apply an upward force to the magnetic beads tethered to Notch receptors on the cell surface, corresponding to 1 magnet per well. The distance vs. magnet force curve was used per Reference [22] to understand force magnitudes. Cells were cultured for 3 days, with or without the magnet. To explore various force magnitudes, the distance between the cell surface and the magnet was increased by dispensing 100 μL Sylgard 184 Silicone Elastomer pre-polymer and the curing agent (20:1) (PDMS, Part A and B, Ellsworth Adhesive Chemical Co., Germantown, WI, USA) mixture per well. The well height can be varied using various volumes of PDMS to achieve an array of force magnitudes (see Figure 5B for a schematic). The PDMS was cured overnight at 37 °C and was sterilized with 70% ethanol at RT for 1 h, followed by FN (5 μg/cm^2^) surface treatment for 1 h. Cells were cultured with bead-bound Jagged1 and the magnet plate. Gene expression levels were determined 3 days later using RT-qPCR.

### 2.6. RNA Isolation and qPCR Analysis

Total RNA was isolated from HCASMC and 10T1/2 2D cell cultures using Trizol Reagent (Thermo Fisher, Ottawa, ON, Canada)), following the manufacturer’s instruction. 1 μg of total RNA was used to synthesize cDNA using M-MLV reverse transcriptase kit (Promega, Madison, WI, USA) using the supplier’s protocol. Real-time quantitative PCR (RT-qPCR) was conducted in 10 μL reaction volumes, using a Chromo4 Real-time C1000 Touch Thermal Cycler (Bio-Rad, Mississauga, ON, Canada). Gene expression of human Jagged1, Notch3, HES1, SM-α-actin (*Acta2*), calponin (*Cnn1*), myosin heavy chain (*Myh11*), and glyceraldehyde- 3-phosphate dehydrogenase (*GAPDH*, reference gene) were determined with iQTM SYBR^®^ Green Supermix (ThermoScientific, Rockford, IL, USA) according to the recommended protocol of the manufacturer. The qPCR reactions were carried out in a CFX96 Real-Time thermal cycler (Bio-Rad, Mississauga, ON, Canada).

### 2.7. Immunofluorescence Microscopy

HCASMCs, or 10T1/2 cells seeded on fibronectin (FN, Santa Cruz Biotech, Dallas, TX, USA, 5 μg/cm^2^) coated cover slides were treated according to the experimental plan. After 3 days, cells were washed with PBS and fixed using a 4% solution of paraformaldehyde (EMD Chemicals, Gibbstown, NJ) for 15 min. Next, cells were permeabilized in 0.5% (*v/v*) Tritonx-100 in PBS for another 15 min and blocked with 5% BAS in PBS-T for 1 h. Blocking solution was aspirated and 100 μL of the appropriate antibodies (mouse anti-Acta2 (1:100), mouse anti-calponin1/2/3 (1:100), rabbit anti-smoothelin, (1:100), mouse anti-Myh11(1:100)) (Santa Cruz Biotech, Dallas, TX, USA) in 5% BSA PBS-T covered in the fridge overnight at 4 °C. Cells were washed, and then, incubated with the corresponding secondary antibody Alexa-488 conjugated goat anti-mouse and Alexa-594 conjugated goat anti-rabbit (1:150) in 5% BSA PBS-T for 1 h. at RT for primary antibody detection. 4′,6-Diamidino-2-phenylindole (DAPI; 300 nmol in PBS) was used to visualize cell nuclei, and F-actin was stained with Alexa™ Fluor 594-conjugated phalloidin (1:100). A LSM 510 confocal microscope (Carl-Zeiss, Toronto, ON, Canada) was used to image the cells. Quantification of protein fluorescence intensity was performed using ImageJ software (version 1.52q, National Institutes of Health, Bethesda, MD, USA), and data was normalized to the control fluorescence expression.

### 2.8. Western Blotting

HCASMCs and 10T1/2 cells seeded on 6-well plates were treated for 3 days as described above. Cells were lysed in 150 μL of ice-cold NP-40 Lysis buffer containing a protease inhibitor cocktail (Roche Diagnostics, Mannheim, Germany), to extract whole cell lysate. Protein concentrations were determined using a Pierce BCA protein 562 nm colorimetric protein assay (Pierce^TM^ 562-nm Protein Assay Kit, ThermoScientific, Rockford, IL, USA) according to the manufacturer’s instructions. An amount of 20–50 μg of protein was loaded and separated by 10% sodium dodecyl sulfate–polyacrylamide gel electrophoresis (SDS-PAGE) for 1 h and, subsequently, transferred onto a nitrocellulose membrane. Transfer efficiency and homogeneous loading were assessed by Ponceau S stain. Membranes were then blocked with 5% non-fat dry milk in 1XPBS for 1 h prior to incubating with primary antibodies: anti-SM α-actin (1:1000 dilution), anti-calponin1/2/3 (1:1000 dilution), anti-Jagged1 (1:200 dilution), anti-Notch3 (1:200 dilution) (all from Santa Cruz Biotech, Dallas, TX, USA), and anti-GAPDH (1:5000 dilution) (Temecula, CA, USA) overnight at 4 °C. The blots were probed with HRP-conjugated secondary antibodies for 1 h followed by the ECL detection (SuperSignal^®^West, Pierce, ThermoScientific, Rockford, IL, USA). Bio-Rad’s ChemiDoc^TM^ XRS+ System was used to image the membranes and blots were quantified using Image Lab software (version 4.1, Bio-Rad, Hercules, CA, USA).

### 2.9. Statistical Analysis

Data are presented as the normalized means to the control sample ± SD. Statistical significance was calculated either using student’s *t*-test or one-way ANOVA followed by Tukey’s post hoc test to compare differences between groups. Values of *p* < 0.05 were considered to be statistically significant.

## 3. Results

### 3.1. The Effect of Bead-Bound Jagged1 as a Surrogate for Endothelial-Bound Jagged1 for Notch Activation in HCASMCs

Since Notch signaling is context-dependent, the presentation of Jagged1 to SMCs is important to control cellular behavior. In this study, we assessed the presentation of Jagged1 in its soluble and bead-immobilized form to stimulate the Notch3 receptor on HCASMCs (Scheme shown in Figure 1A). The Jagged1 binding capacity to Protein G beads was determined using the wash fractions, and HCASMC response was analyzed with increased Jagged1 concentration up to 5 μg (Figure S1). 2.5 μg was sufficient to induce contractile gene expression and used exclusively moving forward. DAPT, a Notch specific inhibitor, prevents cleavage of the NICD, as shown schematically in Figure 1B. This was used to demonstrate Notch cause and effect. Various SMC and Notch signaling markers were probed (Figure 1C–F); *HES1* indicates activation of Notch signaling, *Acta2* and *Cnn1* are early- and mid-stage differentiation markers, and *Myh11* is a late-stage differentiation marker.

Bead-bound but not soluble Jagged1 increased Notch activation as shown by *HES1* expression (Figure 1C). Furthermore, bead-bound Jagged1 caused an upregulation of early/mid-stage smooth muscle-specific markers *Acta2* and *Cnn1,* indicating increased expression of functional contractile SMC markers (Figure 1D,E). This data demonstrated a requirement for tethering of Jagged1 to the bead surface to induce Jagged1-directed Notch signaling in HCASMCs. Cells treated with bead-bound Jagged1 or its soluble form did not respond with increased *Myh11* expression, suggesting *Myh11* might not be regulated by Notch signaling (Figure 1F).

DAPT was used to inhibit Notch signaling and was able to significantly attenuate the bead-bound Jagged1 induced gene response, suggesting a direct cause and effect of Notch signaling and gene expression. VSMC protein expression was consistent with the gene expression data as demonstrated by Western blot (Figure 1G). Protein expression was also used to evaluate the effect of Jagged1 delivery on ligand-receptor expression. Bead-bound Jagged1 upregulated Jagged1 ligand expression in the signal-receiving cell (Figure 1G). Induced expression of Jagged1 in the signal-receiving cell is important to initiate the positive feedback loop and drive signal propagation through lateral induction radially through the vascular wall to pattern the artery wall and control phenotype [34,35].

**Figure 2:**
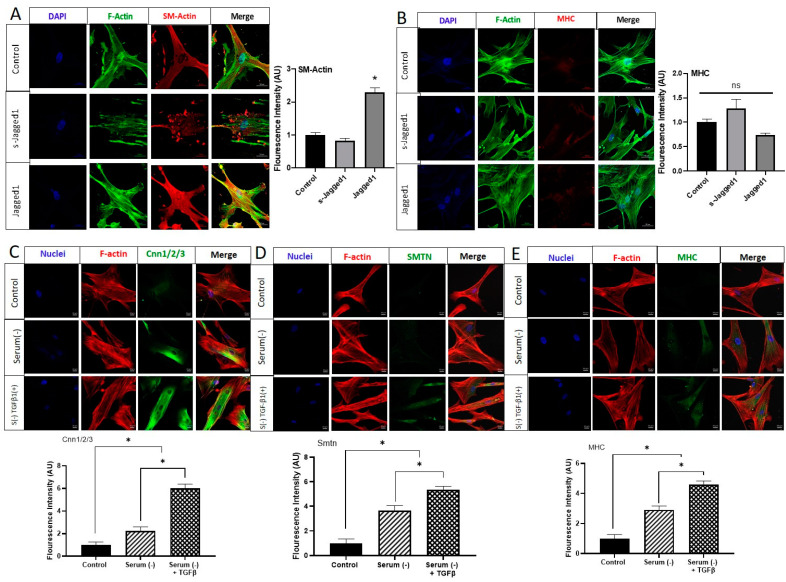
Immunofluorescence microscopy of Jagged1-directed SMC marker protein expression and the effect of serum starvation on late-stage contractile protein expression of HCASMC. Immunofluorescence images comparing the effect of soluble and bead-bound Jagged1 on (**A**) SM-Actin, and (**B**) MHC are shown. Scale bar = 50 μm. The bar graphs shown to the right of Figures A and B are the corresponding integrated density fluorescence quantification presented as mean ± SD (*n* = 5). The control cells in Figures A and B are HCASMCs cultured in SmGM-2. Immunofluorescence staining for the effect of serum starvation and TGFβ1 (2 ng/mL) on the SM-specific protein expression of (**C**) Cnn1/2/3, (**D**) SMTN, and (**E**) MHC. Scale bar = 20 μm. The bar graphs below are the corresponding integrated density fluorescent quantifications presented as mean ± SD (*n* = 15). The control cells are HCASMCs cultured in DMEM supplemented with 5% serum. * indicates significance at *p* < 0.05, ns indicates no statistical significance.

Consistent with the gene expression and Western blot data (Figure 1D,G), the immunofluorescence staining of α-smooth muscle actin (SM-Actin) demonstrated complementary evidence that the use of bead-bound Jagged1 could control SMC phenotype (Figure 2A). Similarly, in agreement with the gene expression data (Figure 1F), bead-bound Jagged1 had no significant influence on myosin heavy chain (MHC) expression (Figure 2B). MHC is an important contractile protein in mature SMCs; therefore, other factors may be required for this late-stage marker expression.

In order to evaluate the expression of the late-stage SMC markers smoothelin (SMTN) and MHC, we tested the role of serum starvation. Serum starvation has previously been shown to induce the phenotypic change in vascular smooth muscle cells from a de-differentiated state to a fully contractile, elongated, and spindle-shaped morphology with elevated myofilament density [36]. To evaluate this, HCASMCs cultured to sub-confluence were serum-starved for 72 h. The addition of 2 ng/mL transforming growth factor-beta (TGFβ1) was also used to further induce a contractile phenotype. As shown in Figure 2C–E, serum-starvation of HCASMCs was able to significantly stimulate protein expression of Cnn1/2/3. Serum-starvation was also able to stimulate SMTN and MHC, which were not achieved with Jagged1 beads alone. When serum starvation was combined with the addition of TGFβ1, another known contractile phenotype inducer, its effect was significantly higher than serum starvation alone. It was evident from these studies that bead-bound Jagged1 alone was not sufficient in driving HCASMCs towards their mature contractile phenotype. Thus, co-presentation of other factors may be needed.

### 3.2. Co-Presentation of Jagged1 and TGFβ1 to Enhance SMC Maturity

**Figure 3:**
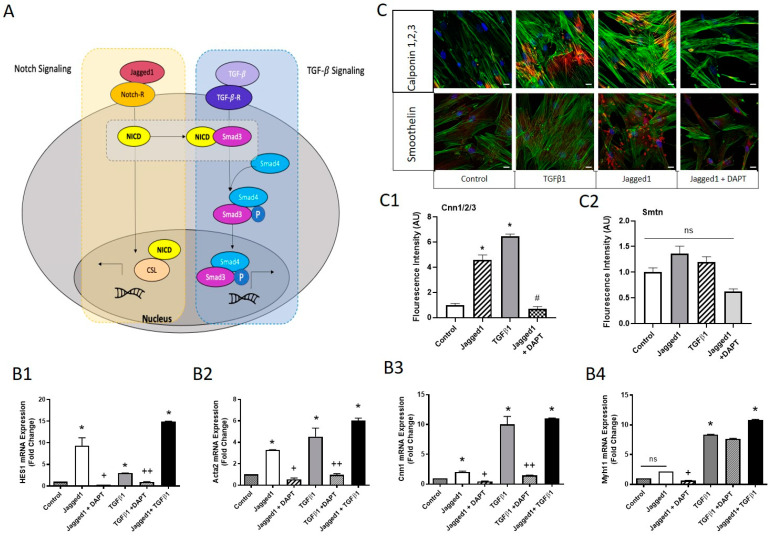
Convergence in Notch and TGFβ signaling induced a mature contractile HCASMC phenotype. (**A**) Scheme for Notch and TGFβ signaling convergence (proposed in the literature, Ref [40]). The NICD co-operatively interacts with Smad 2/3, an intracellular transducer of TGFβ1, influencing the regulation of downstream targets. (**B1**–**B4**) HCASMCs were treated for 3 days with 2.5 μg/mL bead-bound Jagged1 or 2 ng/mL TGFβ1. Cells were also treated in combination with the Notch inhibitor DAPT (described in the methods) for possible signal convergence between Notch and TGFβ1. Gene expression levels of *HES1, Acta2, Cnn1,* and *Myh11* are shown. Data are presented as the normalized mean ± SD, *n* = 6. * Indicates significance from the control cells, + indicates significant downregulation from the Jagged1 treatment group, and ++ indicates significant downregulation from the TGFβ1 treatment group at *p* < 0.05 (ns = not significant). (**C**) Immunofluorescence staining of HCASMCs treated with bead-bound Jagged1 and TGFβ1 stained for calponin 1,2,3 and smoothelin. Scale bar = 50 μm. (**C1**,**C2**) Fluorescence intensity quantifications are presented as the normalized mean to the control ± SD. * indicates significance from the control, and # indicates significant downregulation from the Jagged1 treatment group. Control cells were untreated HCASMCs cultured in SmGM-2. ns indicates no statistical significance.

TGFβ signaling components have been shown to be upregulated at the site of vascular injury, implicated in extracellular matrix accumulation, and VSMC proliferation, migration, and differentiation [37,38]. As demonstrated in Figure 2D,E, late-stage contractile marker expression of SMTN and MHC were upregulated using TGFβ1. Figure 3 shows the effect of bead-bound Jagged1 and soluble TGFβ1 ligands co-presentation on HCASMC phenotype. Due to the prominent response of both ligands in Notch activation, SMC phenotype control, and supporting evidence from literature [15,39], we hypothesized that these ligands could work co-operatively as co-regulators of SMCs. In fact, specific direct protein interactions have linked Notch and TGFβ signaling, by which the NICD acts co-operatively with Smad 2/3 as an intracellular transducer of TGFβ signaling [40] as depicted in Figure 3A.

In view of the above, we investigated the relative *HES1* expression of HCASMCs in response to bead-bound Jagged1 and TGFβ1 (Figure 3B1–B4). While bead-bound Jagged1 enhanced significant Notch activation (*HES1*) when compared to TGFβ1, TGFβ1 alone was still able to activate Notch through *HES1*, hinting at pathway cross-talk. Both Jagged1 and TGFβ1 ligands significantly upregulated SMC contractile markers *Acta2* and *Cnn1*; however, the effect of TGFβ1 was stronger. Notably, TGFβ1 was able to significantly upregulate *Myh11,* which was not seen by bead-bound Jagged1 (Figure 3B4), while increased *Cnn1* expression by both ligands was observed using immunofluorescent imaging (Figure 3C,C1,C2).

A link between Jagged1 and TGFβ1 signaling was established using DAPT, a gamma-secretase inhibitor. DAPT significantly attenuated *HES1*, *Acta2*, and *Cnn1* in HCASMCs cultured with bead-bound Jagged1 or TGFβ1 (Figure 3B1–B3). DAPT inhibits the gamma-secretase cleavage of Notch; thus, to attenuate TGFβ1 signaling, intracellular components such as Smad3 must be affected by the inhibited Notch intracellular domain. Additionally, co-delivery of both ligands had a significantly increased expression of *HES1*, *Acta2*, *Cnn1* and *Myh11* expression, suggesting an additive or synergistic relationship superior to delivery of an individual ligand. Overall, we have demonstrated a combination approach with a high potential to modulate SMC phenotype control.

### 3.3. Notch Activation in Embryonic Multipotent Mesenchymal Cells and Their Differentiation towards a Vascular Smooth Muscle Cell Lineage

**Figure 4:**
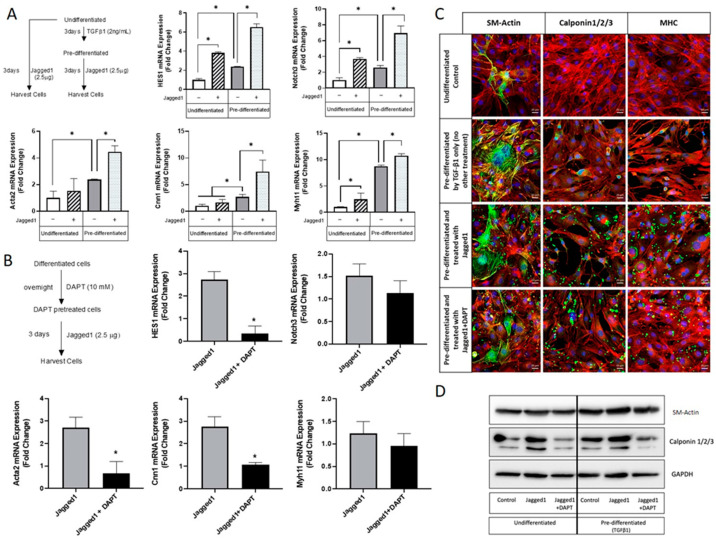
Bead-bound Jagged1 directs maturation of pre-differentiated 10T1/2 progenitor cells to contractile vascular smooth muscle cells. (**A**) Undifferentiated and pre-differentiated 10T1/2 cells were treated with bead-immobilized Jagged1. Gene expression of *HES1, Notch3, Acta2, Cnn1,* and *Myh11* were quantified. Data presented as the mean ± SD (*n* = 3). * indicate significance at *p* < 0.05. (**B**) Pre-differentiated cells were treated with DAPT, and the gene expression levels of *HES1, Notch3, Acta2, Cnn1*, and *Myh11* were quantified. Data presented as the mean ± SD (*n* = 3). * indicates significant attenuation of Jagged1 at *p* < 0.05. (**C**) Immunofluorescence imaging showing the expression levels of SM-Actin, Calponin, and MHC in undifferentiated and pre-differentiated 10T1/2 cells. Scale bar = 20 μm. Red channel: F-Actin, Blue channel: DAPI, Green channel: SM-Actin, calponin1,2,3, and MHC. (**D**) Western blot of Acta2 and Cnn1/2/3 in undifferentiated and pre-differentiated 10T1/2 cells.

The foregoing studies showed the ability of bead-bound Jagged1 to drive cultured HCASMCs towards a fully differentiated contractile phenotype. However, HCASMCs are not readily available for engineering vascular tissues due to their limited proliferation and the difficulty of harvesting autologous cells from patients. As an alternative, progenitor cell lines with a robust growth rate can be used to engineer a model tissue. Although progenitor cell lines from mice cannot be used to engineer a clinically relevant tissue, these cells are useful to study if Jagged1 is involved in the vascular differentiation of progenitor cells. Therefore, we investigated the effects of bead-bound Jagged1 to control vascular smooth muscle differentiation of mouse embryonic multipotent mesenchymal progenitor cells (10T1/2 cells). TGFβ1 has been reported to direct 10T1/2 cells towards a smooth muscle cell lineage [41]. New to the current study is the influence of bead-bound Jagged1 to induce vascular smooth muscle cells. Soluble Jagged1 did not influence 10T1/2 cell vascular linage commitment (Figure S2), again demonstrating a need for its immobilization. The effect of bead-bound Jagged1 was investigated in two different approaches: (i) exposing undifferentiated 10T1/2 cells to bead-immobilized Jagged1, and (ii) pre-differentiating 10T1/2 cells towards a vascular lineage with 2 ng/mL TGFβ1 and then treating them with bead-immobilized Jagged1 to induce a contractile phenotype (Figure 4).

Although bead-bound Jagged1 upregulated *HES1* and the *Notch3* receptor expression in the undifferentiated 10T1/2 cells, indicating Notch3 activation, it failed to upregulate functional SMC genes *Acta2, Cnn1* while only modestly upregulated *Myh11*. In contrast, pre-differentiation of 10T1/2 cells was required to induce robust expression of these SMC markers with significant fold increases (Figure 4A). Thus, prior lineage commitment was needed for bead-bound Jagged1 to be an effective differentiation agent. A Notch inhibitor DAPT was used to show cause and effect (Figure 4B,D). As presented in Figure 4B, the specificity of DAPT is demonstrated by the significant downregulation of Hes1, a downstream target of Notch signaling. In response to DAPT treatment, *Acta2* and *Cnn1* genes were significantly downregulated, suggesting that these genes are downstream targets of the Notch signaling. Consistent with Figure 1F, Figure 2B, and Figure 3B4, *Myh11* was not responsive to Notch activation and DAPT treatment. Since treatment with DAPT did not impact *Myh11,* the underlying mechanism of TGF-β1 associated smooth muscle differentiation may play a dominant role rather than the Notch3 signaling pathway in 10T1/2 cells. Pre-differentiation using TGF-β1 promoted expression levels of the mid- and late-SM-marker proteins calponin1/2/3 and MHC (Figure 4C). Importantly, the addition of bead-immobilized Jagged1 under the pre-differentiated state further enhanced the fluorescence of these SM-markers (Figure 4C). The Western blot data shown in Figure 4D and the corresponding quantification, shown in supplementary Figure S3, were consistent with the gene expression data.

### 3.4. Mechanosensitivity of Jagged1-Notch3 Signaling

**Figure 5:**
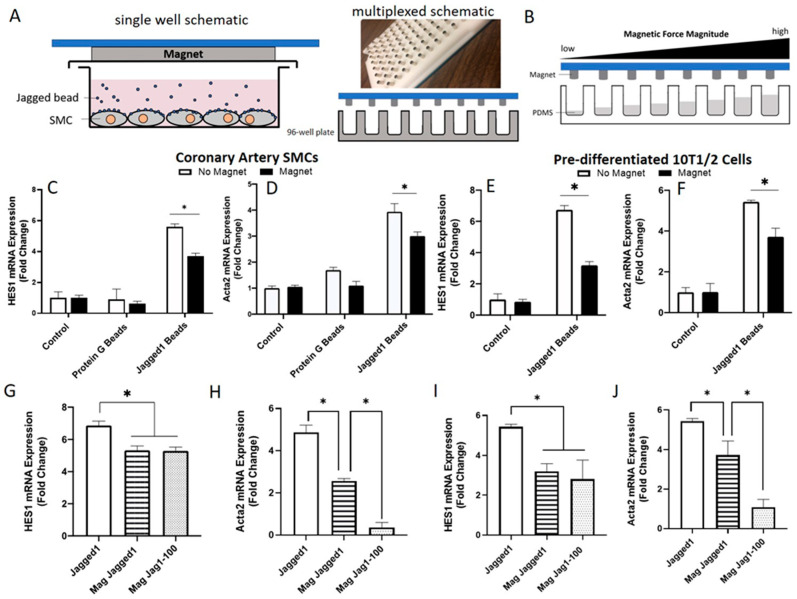
The effect of magnetic tweezer forces in Notch activation. (**A**) Experimental design and set-up. HCASMCs were cultured with Jagged1 bound Protein G beads. A magnetic plate containing 96-cylindrical magnets was positioned over the culture plate with a 1–1 well to magnet ratio to apply a force to the Jagged1 magnetic beads tethered to the Notch3 receptor on HCASMCs or 10T1/2 cells. (**B**) Various volumes of PDMS were dispensed to the wells to create a terraced height between the cell surface and the magnets, where increasing well height corresponds to increased force magnitude. Gene expression of *HES1* and *Acta2* with and without magnets in HCASMCs (**C**,**D**) and pre-differentiated 10T1/2 cells (**E**,**F**). Gene expression of *HES1,* and *Acta2* at two different distances from the HCASMCs surface to the magnets (**G**,**H**) and from the pre-differentiated 10T1/2 cells (**I**,**J**). Data presented as the mean ± SD (*n* = 3), normalized to magnet control. * indicates significance at *p* < 0.05. The different distances from the cell surface to the magnet tips are provided in Figure S4.

Some Notch signaling components are mechanosensitive due to the pulling forces provided by the endocytosis of the ligand by the signal-sending cell following receptor-ligand binding [16,42,43]. Since cell-mediated endocytosis is absent in our system, the effectiveness of bead-bound Jagged1 was explored to gain insight into the role of tension forces for Notch signal propagation. A magnetic tweezer assay was used to provide a tension force (in the pN range) to the ligand-receptor bridge in a multiplexed format, shown in Figure 5A. To control force magnitude (separation distance between the cells and the magnets), the distance from the cell surface to the magnet tip was changed by adding PDMS to the culture wells with the assumption that force magnitude was proportional to height as schematically shown in Figure 5B.

The addition of tension force to the ligand-receptor complex caused a significantly decreased expression of *HES1*, and *Acta2* in both HCASMCs (Figure 5C,D), and pre-differentiated 10T1/2 cells (Figure 5E,F). The magnetic force alone did not show any adverse effect on the cells, nor did the cells negatively respond to the magnet in the presence of Protein G beads (no Jagged1 bound and, hence, no Notch3 binding capability). To explore the effect of increased force magnitude, the distance from the cell surface to the magnets was decreased using PDMS (Figure 5B). An increased force magnitude did not affect *HES1* Notch activation (Figure 5G,I) but further decreased *Acta2* gene expression (Figure 5H,J) in both HCASMCs and 10T1/2 cells. This trend was also seen with other force magnitudes (Figure S4). The applied force magnitude corresponding to PDMS volume is found in Table S1. Therefore, bead-bound Jagged1 alone was sufficient for Notch activation in vascular smooth muscle cells, and external force was not required to control Jagged1-Notch3 signaling in HCASMCs, contrary to what was seen with Dll1 and Dll3 ligands in other cell types [20,21,22].

## 4. Discussion

Endothelial-bound Jagged1 is critical to control the vascular tone, as demonstrated through co-culture studies [11,44]. During disease states or vascular intervention, this signal is often lost due to endothelial cell denudation. Therefore, the delivery of this signal through a biomaterial platform (e.g., stents) could be useful as a therapeutic agent. Delivery of Jagged1 in its soluble form is controversial; it has been suggested to promote cellular differentiation [45,46,47], or act as an inhibitor [48,49,50]. Most research has alluded to the fact that Notch is contact-dependent [51], and contact geometry might be an important mediator [51]. Therefore, it is suggested that for Notch activation and signal propagation, there is a need for anchorage or immobilization of the ligand.

Entrapment of Jagged1 alone in a biomaterial has been shown to be insufficient to induce Notch signaling [52]; however, we have demonstrated that bead-bound Jagged1 can mimic the signaling cell surface by presenting Jagged1 adjacent to HCASMCs, allowing for direct cell-bead contact (Figure 1). The bead-bound affinity immobilization presentation strategy presented is sufficient to induce functional gene response of early-stage contractile markers, but there was a lack of expression of mature SMC markers myosin heavy chain and smoothelin. Given this, we investigated culture conditions and other vascular regulators to improve SMC control. In the native tissue, multiple pathways interact and act as co-regulators to control cell fate decisions. Therefore, it is not surprising that multiple factors co-delivered together are effective in controlling SMC phenotype switching (Figure 3).

There are limitations to the use of HCASMCs for vascular tissue engineering and regenerative medicine strategies as they are not readily available. This led to the investigation of other cell sources, which include, but are not limited to, pluripotent stem cells, bone marrow stem cells, adipose-derived stem cells, and progenitor cell lines [53]. Aside from stem cells, 10T1/2 progenitor cells have demonstrated similar differentiation capability to mesenchymal stem cells and can be differentiated into a vascular lineage using appropriate conditions and factors [54,55]. Here we showed that bead-bound Jagged1 could influence progenitor cell differentiation/maturation towards a vascular lineage, but this was only effective once cells were pre-differentiated using TGFβ1(Figure 4). Immobilized Jagged1 has been used previously for osteoblast [56], and cardiac [57] lineage commitments. We reasoned that without pre-differentiation, 10T1/2 cells in their undifferentiated state might have a higher affinity for differentiation into a non-vascular lineage. Therefore, although Notch is activated, these cells may be undergoing differentiation into other lineages and not towards the intended vascular smooth muscle cell. This alludes to temporal, biphasic effects on Notch activation, which have been demonstrated in cardiac differentiation whereby Jagged1 treatment of undifferentiated embryonic stem cells promoted ectodermal differentiation, and Jagged1 treatment on cardiovascular progenitor cells resulted in cardiac differentiation [58]. Together this highlights the importance of the cell differentiation state and its response to Jagged1 delivery. Once cells were pre-differentiated, bead-bound Jagged1 demonstrated a vascular linage commitment and an enhanced cell maturity, but *Myh11* expression was still lacking. Furthermore, bead-bound Jagged1 alone was inadequate to induce the fully matured *Myh11* marker in HCASMCs. Since we have previously reported that long-term culture combined with physiologic perfusion/pulsatile bioreactor led to the expression of MHC protein by pre-differentiated 10T1/2 cells [59], it may be instructive to culture cells in a scaffold with the presence of Jagged1 for a longer time under dynamic conditions to fully mature them in a Notch-dependent manner.

To improve signal efficacy, aside from immobilization, prior studies proposed the mechanosensitive nature of Notch signaling [60,61,62]. Therefore, to enhance Notch signaling in HCASMCs and 10T1/2 cells, we investigated the effects of a pulling force, which aligns with the Notch “pulling model”. The Notch extracellular domain is suggested to unfold under tension, revealing the site for extracellular domain cleavage and allowing for Notch activation. Therefore, the ligand-receptor complex acts as a force sensor. Our data (Figure 5) suggested that signaling was negatively influenced by a molecular tension force, which is contrary to what has been reported with Delta-like ligands [21,22]. However, from a vascular pathophysiological perspective, the narrowing of vessels increases shear on ECs, which consequently increases strain on SMCs, and given hemodynamic loading effects on EC-SMC signaling [62,63], this response may mimic what is seen in native tissues.

Interestingly, studies have shown a strain responsive decrease for Notch3 expression in VSMCs under uniaxial strain, unlike Notch1 and Notch2 [64]. As for the ligands, the same study [64] demonstrated that Dll1 increased with strain and Jagged1 decreased with strain. Therefore, there is a link by which both Notch3 and Jagged1 expression levels are negatively influenced by strain. Since we used Jagged1-Notch3 pairs in the present study, *Hes1* activation decreased when a magnetic force was applied, which is consistent with the above-cited study. This, in turn, leads to reduced SMC *Acta2* gene expression. Furthermore, vimentin is suggested to be involved for efficient receptor-ligand trans-endocytosis [65]. However, bead-bound Jagged1 alone was able to rescue maturation of SMCs in aortic ring cultures from vimentin knock-out mice, which may indicate that a vimentin-induced pulling force was not needed for SMC control [62]. Thus, extending the context-dependent nature of Notch signaling, the mechanosensitive nature of cells may not always rely on force but instead rely more on specific receptor-ligand pairings. Overall, bead-bound Jagged1 has provided the necessary cues for Notch activation in HCASMCs, which could be due to the ability of Jagged1 to cluster and form dimers [66,67], allowing for limited traction for the pulling force.

## 5. Conclusions

In the current study, we have examined the role of soluble and bead-bound Jagged1 to control HCASMC phenotype and vascular differentiation of progenitor cells. We demonstrated that Jagged1 immobilization is required to induce Notch specific smooth muscle cell markers. We have also shown that 10T1/2 cell pre-differentiation using TGFβ1 towards a vascular lineage was needed before Jagged1-directed cell maturation is effective. Although we were able to induce early-stage SMC markers, Jagged1 signal alone was not sufficient to induce mature contractile makers, myosin heavy chain and smoothelin; hence, other biochemical factors may be needed to improve signaling efficacy. Finally, we demonstrated that a molecular force is not needed for Jagged1-Notch3 activation and reasoned that bead-immobilization alone provided sufficient clustering or traction forces needed for signaling. To mimic the dynamic regulation of Notch signaling in future studies, polymer chemistry could be harnessed to create spacers that would improve biomolecular recognition, ligand accessibility and provide a dynamic behavior. Collectively, our study demonstrated the effectiveness of Jagged1 as a potential target to regulate VSMC and provided support that Jagged1 could be a beneficial ligand to mature engineered vascular tissues or to be considered as a potential candidate for regulating SMC behavior following vascular injuries.

## Data Availability

The data obtained in this study are presented in this manuscript and supporting information. Any additional questions should be directed to the corresponding author.

## Supplementary Materials

The following are available online at https://www.mdpi.com/article/10.3390/cells10082089/s1, Figure S1: Jagged1 binding to Protein G beads in a concentration-dependent manner, Figure S2: The effect of soluble Jagged1 delivery on multipotent 10T1/2 cells, Figure S3: Western blot quantification demonstrating the influence of bead-bound Jagged1 on undifferentiated and pre-differentiated 10T1/2 cells, and Figure S4: Effect of height and force magnitude on SMC response. Table S1. Force magnitude corresponding to PDMS Volume.

## References

[1] Mack J.J., Luisa Iruela-Arispe M. (2018). NOTCH regulation of the endothelial cell phenotype. Curr. Opin. Hematol..

[2] Hofmann J.J., Iruela-Arispe M.L. (2007). Notch signaling in blood vessels: Who is talking to whom about what?. Circ. Res..

[3] Baeten J.T., Lilly B. (2017). Notch Signaling in Vascular Smooth Muscle Cells. Adv. Pharmacol..

[4] Wang W., Campos A.H., Prince C.Z., Mou Y., Pollman M.J. (2002). Coordinate Notch3-Hairy-related Transcription Factor Pathway Regulation in Response to Arterial Injury. J. Biol. Chem..

[5] Davis-Knowlton J., Turner J.E., Turner A., Damian-Loring S., Hagler N., Henderson T., Emery I.F., Bond K., Duarte C.W., Vary C.P.H. (2019). Characterization of smooth muscle cells from human atherosclerotic lesions and their responses to Notch signaling. Lab. Investig..

[6] Rizzo P., Ferrari R. (2015). The Notch pathway: A new therapeutic target in atherosclerosis?. Eur. Heart J. Suppl..

[7] Aquila G., Fortini C., Pannuti A., Delbue S., Pannella M., Morelli M.B., Caliceti C., Castriota F., De Mattei M., Ongaro A. (2017). Distinct gene expression profiles associated with Notch ligands Delta-like 4 and Jagged1 in plaque material from peripheral artery disease patients: A pilot study. J. Transl. Med..

[8] Bray S.J. (2016). Notch signalling in context. Nat. Rev. Mol. Cell Biol..

[9] Lin C.H., Lilly B. (2014). Notch signaling governs phenotypic modulation of smooth muscle cells. Vascul. Pharmacol..

[10] Liu H., Kennard S., Lilly B. (2010). Notch3 expression is induced in mural cells through an autoregulatory loop that requires endothelial-expressed Jagged-1. Vascular.

[11] Bhattacharyya A., Lin S., Sandig M., Mequanint K. (2014). Regulation of Vascular Smooth Muscle Cell Phenotype in Three-Dimensional Coculture System by Jagged1-Selective Notch3 Signaling. Tissue Eng. Part A.

[12] Doi H., Iso T., Sato H., Yamazaki M., Matsui H., Tanaka T., Manabe I., Arai M., Nagai R., Kurabayashi M. (2006). Jagged1-selective notch signaling induces smooth muscle differentiation via a RBP-Jκ-dependent pathway. J. Biol. Chem..

[13] Zohorsky K., Mequanint K. (2020). Designing Biomaterials to Modulate Notch Signaling in Tissue Engineering and Regenerative Medicine. Tissue Eng. Part B Rev..

[14] Abuammah A., Maimari N., Towhidi L., Frueh J., Chooi K.Y., Warboys C., Krams R. (2018). New developments in mechanotransduction: Cross talk of the Wnt, TGF-β and Notch signalling pathways in reaction to shear stress. Curr. Opin. Biomed. Eng..

[15] Luo K. (2017). Signaling cross talk between TGF-β/Smad and other signaling pathways. Cold Spring Harb. Perspect. Biol..

[16] Langridge P.D., Struhl G. (2017). Epsin-Dependent Ligand Endocytosis Activates Notch by Force. Cell.

[17] Narui Y., Salaita K. (2013). Membrane tethered delta activates notch and reveals a role for spatio-mechanical regulation of the signaling pathway. Biophys. J..

[18] Vooijs M., Schroeter E.H., Pan Y., Blandford M., Kopan R. (2004). Ectodomain shedding and intramembrane cleavage of mammalian Notch proteins is not regulated through oligomerization. J. Biol. Chem..

[19] Nandagopal N., Santat L.A., LeBon L., Sprinzak D., Bronner M.E., Elowitz M.B. (2018). Dynamic Ligand Discrimination in the Notch Signaling Pathway. Cell.

[20] Wang X., Ha T. (2013). Defining single molecular forces required to activate integrin and Notch signaling. Nano Lett..

[21] Wang X., Rahil Z., Li I.T.S., Chowdhury F., Leckband D.E., Chemla Y.R., Ha T. (2016). Constructing modular and universal single molecule tension sensor using protein G to study mechano-sensitive receptors. Sci. Rep..

[22] Gordon W.R., Zimmerman B., He L., Miles L.J., Huang J., Tiyanont K., McArthur D.G., Aster J.C., Perrimon N., Loparo J.J. (2015). Mechanical Allostery: Evidence for a Force Requirement in the Proteolytic Activation of Notch. Dev. Cell.

[23] Pedrosa A.R., Trindade A., Fernandes A.C., Carvalho C., Gigante J., Tavares A.T., Diéguez-Hurtado R., Yagita H., Adams R.H., Duarte A. (2015). Endothelial jagged1 antagonizes Dll4 regulation of endothelial branching and promotes vascular maturation downstream of Dll4/Notch1. Arterioscler. Thromb. Vasc. Biol..

[24] Boucher J., Gridley T., Liaw L. (2012). Molecular Pathways of Notch Signaling in Vascular Smooth Muscle Cells. Front. Physiol..

[25] Benedito R., Roca C., Sörensen I., Adams S., Gossler A., Fruttiger M., Adams R.H. (2009). The Notch Ligands Dll4 and Jagged1 Have Opposing Effects on Angiogenesis. Cell.

[26] Gonçalves R.M., Martins M.C.L., Almeida-Porada G., Barbosa M.A. (2009). Induction of notch signaling by immobilization of jagged-1 on self-assembled monolayers. Biomaterials.

[27] Lin J., Lin Y., Su L., Su Q., Guo W., Huang X., Wang C., Lin L. (2017). The role of Jagged1/Notch pathway-mediated angiogenesis of hepatocarcinoma cells in vitro, and the effects of the spleen-invigorating and blood stasis-removing recipe. Oncol. Lett..

[28] Osathanon T., Manokawinchoke J., Sa-Ard-Iam N., Mahanonda R., Pavasant P., Suwanwela J. (2019). Jagged1 promotes mineralization in human bone-derived cells. Arch. Oral Biol..

[29] Putti M., de Jong S.M.J., Stassen O.M.J.A., Sahlgren C.M., Dankers P.Y.W. (2019). A Supramolecular Platform for the Introduction of Fc-Fusion Bioactive Proteins on Biomaterial Surfaces. ACS Appl. Polym. Mater..

[30] Li H., Yu B., Zhang Y., Pan Z., Xu W., Li H. (2006). Jagged1 protein enhances the differentiation of mesenchymal stem cells into cardiomyocytes. Biochem. Biophys. Res. Commun..

[31] Dishowitz M.I., Zhu F., Sundararaghavan H.G., Ifkovits J.L., Burdick J.A., Hankenson K.D. (2014). Jagged1 immobilization to an osteoconductive polymer activates the Notch signaling pathway and induces osteogenesis. J. Biomed. Mater. Res. Part A.

[32] Blache U., Vallmajo-Martin Q., Horton E.R., Guerrero J., Djonov V., Scherberich A., Erler J.T., Martin I., Snedeker J.G., Milleret V. (2018). Notch-inducing hydrogels reveal a perivascular switch of mesenchymal stem cell fate. EMBO Rep..

[33] Beckstead B.L., Santosa D.M., Giachelli C.M. (2006). Mimicking cell–cell interactions at the biomaterial–cell interface for control of stem cell differentiation. J. Biomed. Mater. Res. Part A.

[34] Sjöqvist M., Andersson E.R. (2019). Do as I say, Not(ch) as I do: Lateral control of cell fate. Dev. Biol..

[35] Hoglund V.J., Majesky M.W. (2012). Patterning the artery wall by lateral induction of Notch signaling. Circulation.

[36] Han M., Wen J.K., Zheng B., Cheng Y., Zhang C. (2006). Serum deprivation results in redifferentiation of human umbilical vascular smooth muscle cells. Am. J. Physiol. Cell Physiol..

[37] Tsai S., Hollenbeck S.T., Ryer E.J., Edlin R., Yamanouchi D., Kundi R., Wang C., Liu B., Kent C. (2009). TGF-B through Smad3 signaling stimulates vascular smooth muscle cell proliferation and neointimal formation. Am. J. Physiol. Heart Circ. Physiol..

[38] Low E.L., Baker A.H., Bradshaw A.C. (2019). TGFβ smooth muscle cells and coronary artery disease: A review. Cell Signal..

[39] Kurpinski K., Lam H., Chu J., Wang A., Kim A., Tsay E., Agrawal S., Schaffer D.V., Li S. (2010). Transforming growth factor-β and notch signaling mediate stem cell differentiation into smooth muscle cells. Stem Cells.

[40] Blokzijl A., Dahlqvist C., Reissmann E., Falk A., Moliner A., Lendahl U., Ibáñez C.F. (2003). Cross-talk between the Notch and TGF-β signaling pathways mediated by interaction of the Notch intracellular domain with Smad3. J. Cell Biol..

[41] Dayekh K., Mequanint K. (2020). The effects of progenitor and differentiated cells on ectopic calcification of engineered vascular tissues. Acta Biomater..

[42] Fortini M.E., Bilder D. (2009). Endocytic regulation of Notch signaling. Curr. Opin. Genet. Dev..

[43] Meloty-Kapella L., Shergill B., Kuon J., Botvinick E., Weinmaster G. (2012). Notch Ligand Endocytosis Generates Mechanical Pulling Force Dependent on Dynamin, Epsins, and Actin. Dev. Cell.

[44] Xia Y., Bhattacharyya A., Roszell E.E., Sandig M., Mequanint K. (2012). The role of endothelial cell-bound Jagged1 in Notch3-induced human coronary artery smooth muscle cell differentiation. Biomaterials.

[45] Kibbie J., Teles R.M.B., Wang Z., Hong P., Montoya D., Krutzik S., Lee S., Kwon O., Modlin R.L., Cruz D. (2016). Jagged1 Instructs Macrophage Differentiation in Leprosy. PLoS Pathog..

[46] Savary E., Sabourin J.C., Santo J., Hugnot J.P., Chabbert C., Van De Water T., Uziel A., Zine A. (2008). Cochlear stem/progenitor cells from a postnatal cochlea respond to Jagged1 and demonstrate that notch signaling promotes sphere formation and sensory potential. Mech. Dev..

[47] Aho S. (2004). Soluble form of Jagged1: Unique product of epithelial keratinocytes and a regulator of keratinocyte differentiation. J. Cell. Biochem..

[48] Sun J., Luo Z., Wang G., Wang Y., Wang Y., Olmedo M., Morandi M.M., Barton S., Kevil C.G., Shu B. (2018). Notch ligand Jagged1 promotes mesenchymal stromal cell-based cartilage repair. Exp. Mol. Med..

[49] Caolo V., Schulten H.M., Zhuang Z.W., Murakami M., Wagenaar A., Verbruggen S., Molin D.G.M., Post M.J. (2011). Soluble jagged-1 inhibits neointima formation by attenuating notch-herp2 signaling. Arterioscler. Thromb. Vasc. Biol..

[50] Urs S., Turner B., Tang Y., Rostama B., Small D., Liaw L. (2012). Effect of soluble Jagged1-mediated inhibition of Notch signaling on proliferation and differentiation of an adipocyte progenitor cell model. Adipocyte.

[51] Shaya O., Binshtok U., Hersch M., Rivkin D., Weinreb S., Amir-Zilberstein L., Khamaisi B., Oppenheim O., Desai R.A., Goodyear R.J. (2017). Cell-Cell Contact Area Affects Notch Signaling and Notch-Dependent Patterning. Dev. Cell.

[52] Dayekh K., Mequanint K. (2020). Comparative Studies of Fibrin-Based Engineered Vascular Tissues 2 and Notch Signaling from Progenitor Cells 1 3. ACS Biomater. Sci. Eng..

[53] Bajpai V.K., Andreadis S.T. (2012). Stem Cell Sources for Vascular Tissue Engineering and Regeneration. Tissue Eng. Part B Rev..

[54] Kiros S., Lin S., Xing M., Mequanint K. (2020). Embryonic Mesenchymal Multipotent Cell Differentiation on Electrospun Biodegradable Poly(ester amide) Scaffolds for Model Vascular Tissue Fabrication. Ann. Biomed. Eng..

[55] Hirschi K.K., Rohovsky S.A., D’Amore P.A. (1998). PDGF, TGF-β, and heterotypic cell-cell interactions mediate endothelial cell-induced recruitment of 10T1/2 cells and their differentiation to a smooth muscle fate. J. Cell Biol..

[56] Ndong J.D.L.C., Stephenson Y., Davis M.E., García A.J., Goudy S. (2018). Controlled JAGGED1 delivery induces human embryonic palate mesenchymal cells to form osteoblasts. J. Biomed. Mater. Res. Part A.

[57] Boopathy A.V., Che P.L., Somasuntharam I., Fiore V.F., Cabigas E.B., Ban K., Brown M.E., Narui Y., Barker T.H., Yoon Y.S. (2014). The modulation of cardiac progenitor cell function by hydrogel-dependent Notch1 activation. Biomaterials.

[58] Tung J.C., Paige S.L., Ratner B.D., Murry C.E., Giachelli C.M. (2014). Engineered Biomaterials Control Differentiation and Proliferation of HumanEmbryonic-Stem-Cell-Derived Cardiomyocytes via Timed Notch Activation. Stem Cell Rep..

[59] Lin S., Mequanint K. (2017). Bioreactor-induced mesenchymal progenitor cell differentiation and elastic fiber assembly in engineered vascular tissues. Acta Biomater..

[60] Seo D., Southard K.M., Kim J., Cheon J., Gartner Z.J., Jun Y., Seo D., Southard K.M., Kim J., Lee H.J. (2016). A Mechanogenetic Toolkit for Interrogating Cell Signaling in Space and Time. Cell.

[61] Mack J.J., Mosqueiro T.S., Archer B.J., Jones W.M., Sunshine H., Faas G.C., Briot A., Aragón R.L., Su T., Romay M.C. (2017). NOTCH1 is a mechanosensor in adult arteries. Nat. Commun..

[62] van Engeland N.C.A., Suarez Rodriguez F., Rivero-Müller A., Ristori T., Duran C.L., Stassen O.M.J.A., Antfolk D., Driessen R.C.H., Ruohonen S., Ruohonen S.T. (2019). Vimentin regulates Notch signaling strength and arterial remodeling in response to hemodynamic stress. Sci. Rep..

[63] Van Engeland N.C.A., Pollet A.M.A.O., Den Toonder J.M.J., Bouten C.V.C., Stassen O.M.J.A., Sahlgren C.M. (2018). A biomimetic microfluidic model to study signalling between endothelial and vascular smooth muscle cells under hemodynamic conditions. Lab Chip.

[64] Loerakker S., Stassen O.M.J.A., Fleur M., Boareto M., Bouten C.V.C. (2018). Mechanosensitivity of Jagged—Notch signaling can induce a switch-type behavior in vascular homeostasis. Proc. Natl. Acad. Sci. USA.

[65] Antfolk D., Sjöqvist M., Cheng F., Isoniemi K., Duran C.L., Rivero-Muller A., Antila C., Niemi R., Landor S., Bouten C.V.C. (2017). Selective regulation of Notch ligands during angiogenesis is mediated by vimentin. Proc. Natl. Acad. Sci. USA.

[66] Andersson E.R., Sandberg R., Lendahl U. (2011). Notch signaling: Simplicity in design, versatility in function. Development.

[67] Ong C.T., Cheng H.T., Chang L.W., Ohtsuka T., Kageyama R., Stormo G.D., Kopan R. (2006). Target selectivity of vertebrate notch proteins: Collaboration between discrete domains and CSL-binding site architecture determines activation probability. J. Biol. Chem..

