# Supplementary material for: Immobilization of Jagged1 Enhances Vascular Smooth Muscle Cells Maturation by Activating the Notch Pathway
Source: Cells. 2021 Aug 14;10(8):2089. doi: 10.3390/cells10082089 (PMC8391929; doi:10.3390/cells10082089)
Supplement: Supplementary file 1 [file cells-10-02089-s001.zip › cells-1284374-supplementary.pdf]

cells

MDPI

Article

Immobilization of Jagged1 Enhances Vascular Smooth Muscle Cells Maturation by Activating the Notch Pathway

Kathleen Zohorsky[1], Shigang Lin[2] and Kibret Mequanint [1,2*]

[1] School of Biomedical Engineering, University of Western Ontario; 1151 Richmond Street, London, N6A 5B9, Canada; kzohorsk@uwo.ca (KZ); kmequani@uwo.ca (KM)

[2] Department of Chemical and Biochemical Engineering, University of Western Ontario; 1151 Richmond Street, London, N6A 5B9, slin45@uwo.ca (SL); kmequani@uwo.ca (KM)

* **Correspondence:** Correspondence: kmequani@uwo.ca (Kibret Mequanint)

A

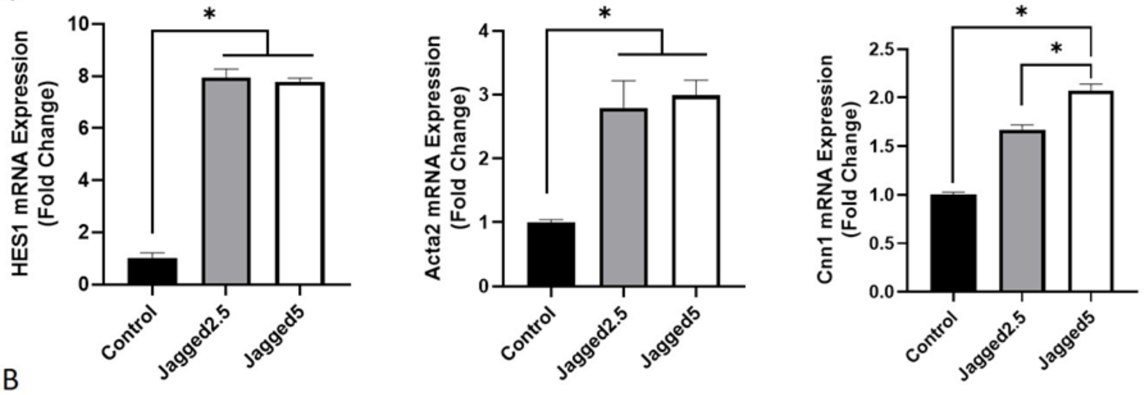

B

Binding Efficiency				
Jagged1 Concentration	Absorbance	Bead Volume (µL)	Total Protein in Wash Fraction (µg)	Total Protein Bound (µg)
Jagged 0	0	20	0	0
Jagged 2.5	0.0055	20	0.174	2.326
Jagged 5	0.0405	20	1.285	3.715

Figure S1. Jagged1 binding to ProteinG beads in a concentration-dependent manner.Recombinant human Jagged1 protein was bound to ProteinG magnetic Dynabeads as described in Methods, through an affinity immobilization scheme. Jagged1 concentrations of 0–5µg were added to 20µL ProteinG beads (in the original bead concentration, 30mg/mL) for 10 min under rotation. **A)** Jagged1 beads (200 beads/cell) were cultured with HCASMCs for 3 days and gene expression of *HES1, Acta2*, and *Cnn1* were investigated using RT-qPCR. Increased protein loading from 2.5 µg to 5 µg did not drastically change HCASMC response. Data is normalized to the control (untreated HCASMCs using SmGM-2). The asterisks indicate significance $p<0.05$. **B)** The wash fractions after Jagged1 incubation were recovered and quantified using an ELISA assay using an absorbance *vs.* concentration standard curve. Total protein in the wash fraction was subtracted from the initial protein loading to quantify total protein bound to the bead fraction. 5 µg was chosen as the upper limit per the manufacturer's instructions for the maximum binding capacity of the selected bead volume.

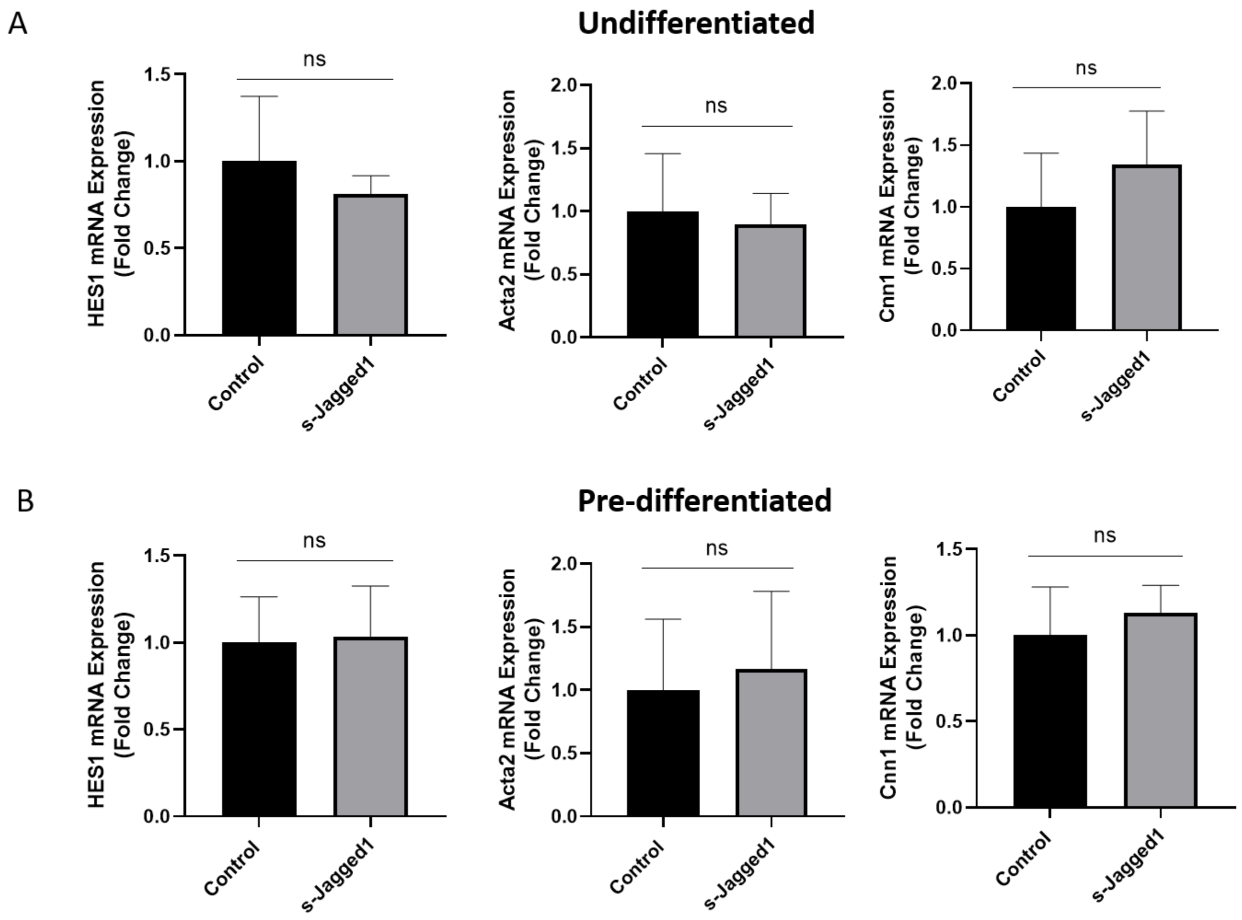

Figure S2. The effect of soluble Jagged1 on multipotent 10T1/2 cells. 10T1/2 cells were cultured for 3 days in DMEM (as specified in Methods) with 2ng/mL TGFβ1 to pre-differentiate the cells into a vascular lineage. **A)** Undifferentiated and **B)** pre-differentiated 10T1/2 cells were treated with soluble Jagged1 at a concentration of 2.5 μg/mL for 3 days. Gene expressions of *HES-1, Acta2,* and *Cnn1* were used to demonstrate Notch specific vascular differentiation. Neither condition responded to Jagged1 delivered in its soluble form. Thus, soluble Jagged1 was insufficient to activate the Notch transcription factor, HES1 and the SMC cell lineage genes in 10T1/2 cells. The data is represented as a normalized mean ± SD. Data is normalized to the control cells. Controls are undifferentiated 10T1/2 cells. No statistical significance was seen as indicated by "ns" (no significance) with $p<0.05$.

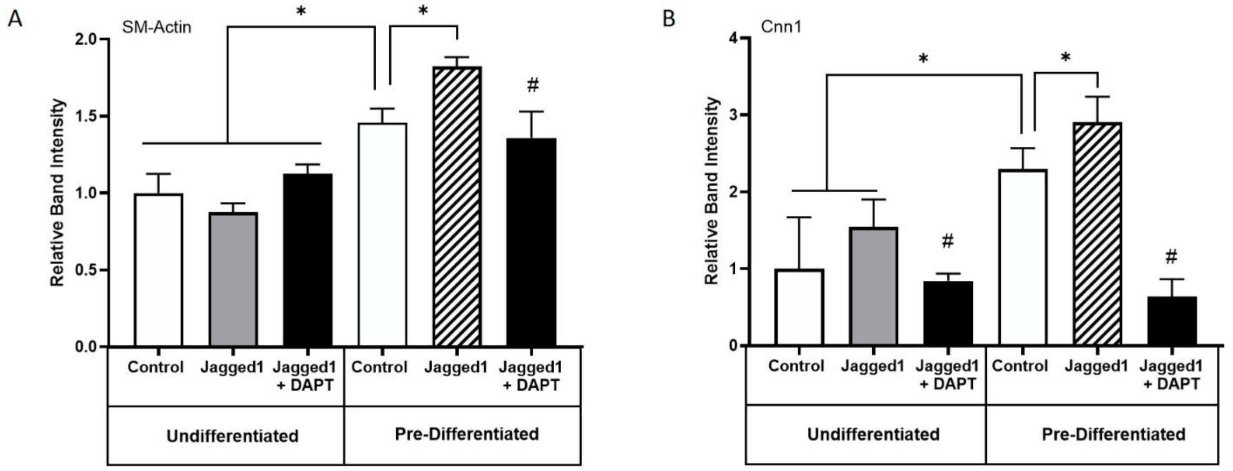

Figure S3. Western blot quantification demonstrating the influence of bead-bound Jagged1 on undifferentiated and pre-differentiated 10T1/2 cells.The relative band intensity of the Western Blot images from Figure 4D are shown above. Quantification of **A)** SM-Actin (42 kDa) and **B)** Cnn1 (34 kDa, the top band of Cnn1/2/3) was performed. Pre-differentiation of 10T1/2 cells with TGFβ1 to induce lineage commitment was required for bead-bound Jagged1 to be an effective contributor

to smooth muscle cell differentiation. Data is presented as the relative mean ± SD to the undifferentiated 10T1/2 cell control. Data was scaled based on the GAPDH loading control for each lane. * indicates significant upregulation from the indicated sample, and # indicates significant downregulation from Jagged1 in the same differentiation state at $p < 0.05$.

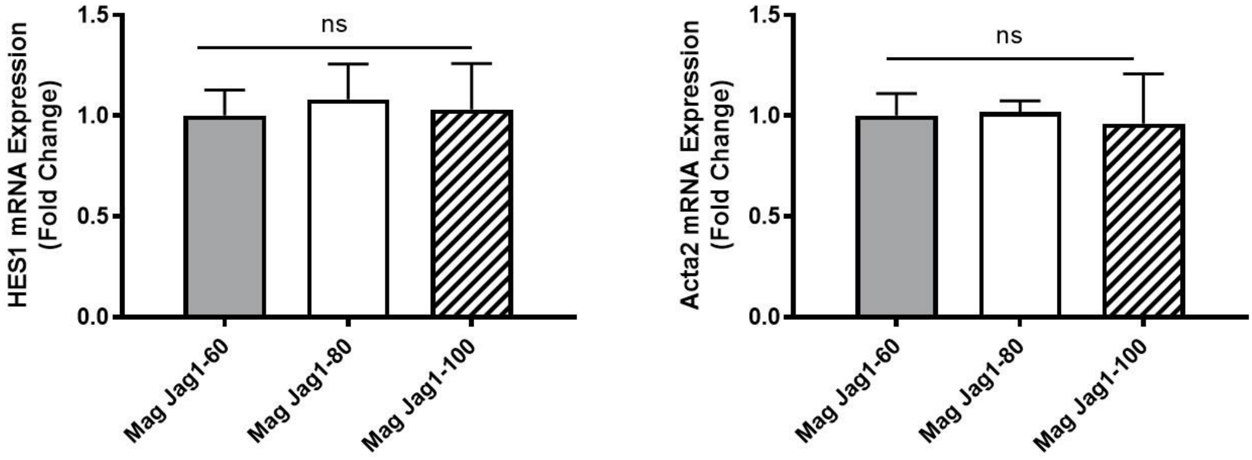

Figure S4. The effect of height (distance from the cell surface to the magnet tip) and force magnitude on SMC response. 60-100 µL of PDMS was dispersed incrementally to create a terraced height between the cell surface and magnets to analyze the effects of various force magnitudes on HCASMCs response. PDMS was cured overnight, sterilized with ethanol, and treated with fibronectin to enhance adherence to the substrate. HCASMCs were cultured on the PDMS surface with the presence of bead-bound Jagged1. A magnetic plate containing 96-cylindrical magnets was positioned over the plate with a 1-1 well to magnet ratio to apply a force to the Jagged1 magnetic beads tethered to the Notch3 receptor on HCASMCs. An increased PDMS volume corresponds to a decreased height to the magnet, which creates higher force magnitudes (**Table S1.**). Gene expressions of *HES1*, *Acta2* were analyzed. The variations of force magnitude at this scale did not change the relative response of cells. The data is presented as a normalized mean ± SD. Data is normalized to Mag Jag1-60.

Table S1. Force magnitude corresponding to PDMS Volume.

PDMS Volume (µL)	Distance (mm)	~Force (pN) *
0	5.2	0.4
60	3.4	0.9
80	2.8	1.1
100	2.2	1.4

*Force values were obtained from the calibration curve of Ref 1.

Reference:

1. Gordon, W.R.; Zimmerman, B.; He, L.; Miles, L.J.; Huang, J.; Tiyanont, K.; McArthur, D.G.; Aster, J.C.; Perrimon, N.; Loparo, J.J.; et al. Mechanical Allostery: Evidence for a Force Requirement in the Proteolytic Activation of Notch. *Dev. Cell* **2015**, *33*, 729–736, doi:10.1016/j.devcel.2015.05.004.
